# Are We Ready for the Use of Foxp3^+^ Regulatory T Cells for Immunodiagnosis and Immunotherapy in Kidney Transplantation?

**DOI:** 10.1155/2012/397952

**Published:** 2012-05-29

**Authors:** Francisco Salcido-Ochoa, Nurhashikin Yusof, Susan Swee-Shan Hue, Doreen Haase, Terence Kee, Olaf Rotzschke

**Affiliations:** ^1^Tregs and HLA Research Force, Department of Renal Medicine, Singapore General Hospital, Outram Road, Singapore 169608; ^2^Tregs and HLA Research Force, Department of Pathology, Singapore General Hospital, Outram Road, Singapore 169608; ^3^Singapore Immunology Network, 8A Biomedical Grove Level 04-06, Immunos Building, Singapore 138648; ^4^Department of Renal Medicine, Singapore General Hospital, Outram Road, Singapore 169608

## Abstract

The existence of T-cell subsets naturally committed to perform immunoregulation has led to enthusiastic efforts to investigate their role in the immunopathogenesis of transplantation. Being able to modulate alloresponses, regulatory T cells could be used as an immunodiagnostic tool in clinical kidney transplantation. Thus, the measurement of Foxp3 transcripts, the presence of regulatory T cells in kidney biopsies, and the phenotypic characterisation of the T-cell infiltrate could aid in the diagnosis of rejection and the immune monitoring and prediction of outcomes in kidney transplantation. Interestingly, the adoptive transfer of regulatory T cells in animal models has been proven to downmodulate powerful alloresponses, igniting translational research on their potential use as an immunomodulatory therapy. For busy transplant clinicians, the vast amount of information in the literature on regulatory T cells can be overwhelming. This paper aims to highlight the most applicable research findings on the use of regulatory T cells in the immune diagnosis and potential immunomodulatory therapy of kidney transplant patients. However, can we yet rely on differential regulatory T-cell profiles for the identification of rejection or to tailor patient's immunosuppression? Are we ready to administer regulatory T cells as inductive or adjunctive therapy for kidney transplantation?

## 1. Introduction

The avoidance of long-term immunosuppression by achieving immunological tolerance would be the ultimate solution to improving long-term patient survival and giving kidney transplant patients a better quality of life. Unfortunately, owing to its complex immunopathogenesis, true immunological tolerance to avert alloresponses has been difficult to achieve. In particular, once the alloresponse is established, it is extremely difficult to control because of its strong and self-amplifying effector mechanisms. These obstacles form the platform of unceasing battles against transplant rejection.

 Amongst the mechanisms implicated in the generation and/or maintenance of immune tolerance, the immunoregulatory role of regulatory T cells (Tregs) is one of the most attractive yet elusive one. In the early 1970s, seminal experiments by Gershon and Kondo [1, 2] unveiled the existence of a population of suppressor T cells, but subsequent failures to substantiate their theory had led to the demise of their idea for almost three decades [3, 4]. The interest in the suppressor T cell resurged in 1995 after Sakaguchi's work, which elegantly demonstrated the existence of a subset of CD4^+^CD25^+^ T cells that appeared to be naturally committed to perform immunoregulation [5]. The expression of the forkhead box transcription repressor factor (Foxp3) was later found to be characteristic of Tregs [6–8], and their designation was changed to Foxp3^+^ Tregs.

 Given the vast evidence demonstrating the contribution of Tregs regulating immune responses in different animal models and clinical situations of autoimmunity and transplantation, great hopes have flourished on the potential use of Tregs as markers of tolerance, transplant rejection, or prediction of graft outcomes. Similarly, great efforts have been put to develop protocols for the use of Tregs as an immunomodulatory therapy in autoimmunity, allergy, and transplantation. In this comprehensive review, after a few notes on Treg biology, we have highlighted the most important research findings on the use of Tregs in the immune diagnosis in kidney transplantation, mainly, based on histopathological evidence of rejection. We attempt to draw our conclusions based on the design quality and results of the available studies. However, with respect to the use of Tregs as immunotherapy in kidney transplantation, the data is still scarce.

## 2. Characterisation of Regulatory T Cells

### 2.1. Origin and Types of Regulatory T Cells

Tregs consist of a heterogeneous population of T cells with the ability of suppressing immune responses. The so-called natural Tregs, or nTregs, are derived from the thymus [5], while the Tregs that develop in the periphery during an adaptive immune response are referred to as induced Tregs, or iTregs. Although both are T cell subsets with regulatory properties, nTregs and iTregs appear to have major differences with respect to their developmental pathways, T-cell receptor (TCR) repertoires [9], as well as activation requirements [10]. It is also likely that they are segregated into different compartments for their effector functions.

 nTregs develop within the thymic medulla, around Hassal's corpuscles, under the influence of both interleukin- (IL-) 2 and tumour growth factor (TGF)*β* [11, 12]. Signalling derived from TCR engagement by major histocompatibility complex (MHC) molecules loaded with self-peptides appears to be crucial for their development, as suggested by the severe depletion of intrathymic Tregs observed upon disruption of proximal TCR signalling by targeted mutations [13]. After exiting the thymus into the periphery, nTregs comprise about 5–10% of the total peripheral T cells [14]. nTregs appear to be a stable population fully and naturally committed to immunoregulation, and their main role is thought to contribute to the maintenance of peripheral tolerance and to prevent the development of autoimmunity.

 On the other hand, iTregs develop in the periphery during an adaptive immune response under the influence of different cues given by the immune system. In particular, a milieu rich in IL-2 and TGF*β* appears to polarise the naïve CD4^+^ T cells towards the iTreg differentiation pathway [15]. Compared to nTregs, iTregs appear to display larger phenotypic plasticity, with the capacity to transform to other subtypes of T cells, depending on the prevailing cytokine milieu. Plausibly, this plastic property of iTregs may serve a physiological role in the immune system by fine-tuning T-cell responses according to the requirements of the immune response. Alternatively, iTregs could be an inherently unstable population by nature, or simply represent a self-regulating, transient, anergic state of recently activated, or overstimulated T cells.

### 2.2. Demethylation at the Foxp3 Gene as a Marker of Authenticity of Regulatory T Cells

The phenotypic plasticity displayed by iTregs, when compared to nTregs, may be owing to the relatively unstable expression of Foxp3 in the former population, which in turn could be partially attributable to the epigenetic differences in the Foxp3 gene in these two Treg subsets [16]. Foxp3 gene is demethylated at the so-called Treg-specific demethylated region (TSDR) in nTregs, but is methylated in iTregs [17]. Interestingly, demethylation at the TSDR on the Foxp3 gene has been proposed to be a hallmark characteristic of authentic Tregs, differentiating them from recently activated conventional T cells [17, 18], which can also express Foxp3 transiently upon activation [19]. However, unlike nTregs, Foxp3 gene is methylated at the TSDR in activated T cells. In fact, Bestard et al. observed that the staining of Foxp3, taking as a surrogate marker for the presence of Tregs, correlated well with the detection of demethylation at the TSDR on the Foxp3 gene on their protocol biopsies [18]. Therefore, they proposed that the analysis of the methylation status at the Foxp3 gene TSDR can differentiate genuine Tregs from recently activated conventional T cells. The authors concluded that a more precise delineation of the true phenotype of the infiltrating T cells by methylation status in a kidney transplant biopsy is likely to better reflect the true clinical significance and potential diagnostic and prognostic implications of Tregs. Further larger studies are required to validate the usefulness of assessing the methylation status at the TSDR in clinical transplantation, although it is promising that this novel technique could potentially add value to the Banff classification.

Although the analysis of the methylation status of the Foxp3 gene at the TSDR appears so far to be the most specific method for the detection of genuine nTregs, it cannot distinguish iTregs from recently activated non-Tregs, as both populations appear to have methylated Foxp3 gene at the TSDR. The difficulty of accurately separating these two T-cell populations with completely different function may have had led to erroneous conclusions drawn in many previous studies. This molecular predicament may also explain why in some studies the detection of Tregs and/or Foxp3 transcripts did not correlate with outcomes (see below), as it is possible that the Foxp3-expressing T cells were not authentic Tregs but simply activated conventional T cells.

### 2.3. Phenotype of Regulatory T Cells

One of the difficulties in the detection and/or isolation of Tregs in peripheral blood is the absence of a specific surface marker. Many surface markers such as CD25, CD39, CD73, CD103, CD134, CD152 (cytotoxic T lymphocyte antigen (CTLA)-4), CD196, and CD357 have been identified on Tregs [20–26]; however, they are not specific for Tregs as these markers can be expressed on activated effector T cells (Teffs) or other cell populations. Although the expression of Foxp3 appears to be the most reliable signature of both nTregs and iTregs [11], it is not expressed on the cell membrane, which precludes its use as a selection marker for isolation. Nonetheless, Foxp3 appears to be pivotal for the development of Treg phenotype and their function [6–8], hence, the detection of Foxp3 expression or transcripts is employed by some investigators as a surrogate marker for the presence or the involvement of Tregs in immunoregulation (see below). Sakaguchi's team has suggested that the differential expression of certain surface, cytoplasmic and nuclear markers, in particular CD45RA, CD25, CD152, and Foxp3, can help to differentiate resting Tregs (CD45RA^+^CD25^+^CD152^lo^Foxp3^lo^) from the highly suppressive activated Tregs (CD45RA^−^CD25^hi^CD152^hi^Foxp3^hi^) [27]. However, for a more precise delineation, assessment of their proliferative state, production of inhibitory cytokines, in vitro suppressive capacity, and analysis of Foxp3 gene methylation appear to be also important.

### 2.4. Suppressive Mechanisms Deployed by Regulatory T Cells

Tregs, similar to conventional T cells, appear to require antigen-specific TCR ligation for their activation, however their suppressive capacities appear to be non-antigen-specific [28]. They have been shown to be able to inhibit T helper (Th)1 cells, Th2 cells, Th17 cells, dendritic cells (DCs), natural killer cells, B cells, and cytotoxic T lymphocytes (CTLs) [28–31], all of which are known effectors of allograft damage. Mechanistically, in several experimental models, they were found to inhibit several components of an immune response, including IL-2 transcription in Teffs, interferon (IFN)*γ* secretion by CTLs as well as DC function [28, 32, 33]. In addition, they appeared to be equipped with suppressive cytokines (such as TGF*β*) on their surface for immunosuppression, have the capacities to induce apoptosis of Teffs, and to degrade inflammatory extracellular adenosine triphosphate (ATP) [28, 31, 34, 35].

 Like conventional Teffs, Tregs also differentiate into effector/memory cells upon antigen-contact [31]. Naïve-type Tregs have been shown to express CD62L and CD197 (chemokine receptor CCR7) [36], which can help them to home primarily to secondary lymphoid organs, where they could potentially modulate the interaction of naïve T cells with antigen-presenting cells (APCs) to prevent excessive stimulation of T cells, or to control their differentiation. In contrast to naïve-type Tregs, effector/memory-like Tregs (T_REM_) appear to suppress inflammation directly inside the tissue, including the kidney allograft. The T_REM_ cells seem to be the natural counterplayers of Th17 cells, an effector/memory subset involved in the propagation of proinflammatory responses. Remarkably, both the T_REM_ and the Th17 cells share the expression of the chemokine receptor CD196, suggesting colocalisation in sites of immune inflammation. Recent studies propose that ATP catabolising enzymes such as CD39 and CD73 expressed on the surface of T_REM_ cells may play a major role in this process [23, 24]. We and other authors have proposed that the expression of CD39 together with other surface markers helps to facilitate the detection of Tregs, particularly the T_REM_ subset. CD39, being an ATPase, removes extracellular ATP released during tissue injury, which is known to be an important mediator of inflammation acting on purinergic receptors and then inducing the secretion of IL-1 [37]. Therefore, ATP catabolism by the T_REM_ subset through CD39 during transplant rejection could be one of the key mechanisms employed by Tregs to control progressive injury [38]. Since the T_REM_ cells have been suggested to manifest their regulatory functions after infiltrating the tissues, it might be more relevant to locate their presence in the kidney transplant T cell infiltrates rather than the general identification of naïve Tregs or Foxp3 transcripts. Our groups are at present investigating the potential immunodiagnostic and immune predictive value of the detection of T_REM_ cells in clinical kidney transplantation.

## 3. Role of Regulatory T Cells in Transplantation

It is now a widely held belief that Tregs have a key role in the immune regulation of transplant rejection. There is a vast amount of data in the literature demonstrating the importance of Tregs in several animal models of transplantation [39–42]. However, most of these are models of skin, pancreatic islets, or heart transplantation. Data from the latter model might resemble more accurately the transplant kidney setting as both kidneys and hearts are vascularised organs. Interestingly, in many models of transplantation, functional Tregs were detected within tolerated grafts [43, 44]. For example, in an elegant murine model of skin graft tolerance [43], the intragraft Tregs were able to transfer the tolerant state to other animals if the tolerated skin was re-transplanted to naïve animals. This suggested that Tregs can actively operate their immunoregulation properties not only in the secondary lymphoid tissues, but also inside the graft directly where the alloantigens and the immunoaggressive cells (i.e., Th1 cells, Th17 cells, and CTLs) interact. Plausibly, Tregs could be recruited and activated inside the graft, where they play an important modulatory role in silencing the effector destructive responses, by arresting the proliferation of Teffs, as well as “prohibiting” their cytokine production capacity, rendering them anergic, as well as inducing their apoptosis, so as to prevent excessive inflammation and collateral damage. It has been postulated that the most crucial factor to the outcome of an allograft is the balance between the ratio of Teffs versus Tregs, either in numbers or in overall functional predominance. However, the factors that underlie the balance between these antagonistic responses are still poorly understood.

## 4. Regulatory T Cells and Immune Tolerance in Clinical Kidney Transplantation

### 4.1. Regulatory T Cells as Markers of “Operational” Tolerance in Kidney Transplantation

The observation of a small subset of kidney transplant patients that spontaneously accepted their grafts in the absence of immunosuppression, the so-called “operational tolerance” has given us hope in our search for the “holy-grail” in transplantation tolerance. Whether Tregs are part of that “holy-grail” is still unknown and continues to puzzle us.

In a cross-sectional analysis comparing the numbers of circulating Tregs among normal subjects, patients undergoing chronic rejection, and patients tolerant to their kidney grafts [45], the authors found that the patients undergoing chronic rejection had lower levels of CD4^+^CD25^+^ Tregs and Foxp3 transcripts, and higher numbers of CD4^+^ T cells with cytotoxic phenotype than the other two groups. In addition, the patients with tolerated kidney grafts displayed similar number of blood Foxp3^+^ Tregs when compared to healthy subjects. From their observations, it cannot be concluded that the Tregs were responsible for the different clinical outcomes, and perhaps the different numbers in Tregs between the patients chronically rejecting their grafts and the tolerant ones could simply be related to the presence, absence, or type of immunosuppression. Nevertheless, the existence of kidney transplant patients spontaneously tolerating their grafts has sustained a series of enthusiastic investigations searching for clues to decipher the secrets of transplantation tolerance. A comparative assessment of the relative function and phenotype of the circulating Tregs in those groups of patients would have been desirable, but it was not performed. Moreover, a phenotypic characterisation of the T cell infiltrates in biopsies of the tolerated kidneys in these patients may be more informative, though not sensible from the ethical point of view as explained by the authors.

By the use of microarray analysis, the same group subsequently demonstrated the expression of a particular set of 49 genes in the tolerant patients not expressed in other patients [46], including Foxp3 and genes regulated by TGF*β*, which is an important cytokine for Treg development and function. They proposed that these genes could be potential biomarkers for tolerance achievement in kidney transplantation. Although their results require further validation, this has nevertheless fueled more interest in the pursuit of transplantation tolerance.

### 4.2. Induction of “Operational” Tolerance in Clinical Kidney Transplantation

Research performed under the auspices of the immune tolerance network has revealed long-term donor-specific unresponsiveness up to 3 years of followup in a small case series of 5 patients that underwent combined kidney and bone marrow transplantation (BMT). These patients were subjected to an immunoablative protocol consisting of anti-CD2 monoclonal antibody (mAb) and cyclophosphamide with or without rituximab and thymic irradiation to facilitate engraftment and attempting to induce “operational” kidney transplant tolerance [47]. Interestingly, the tolerant patients displayed an increment in Treg numbers during the lymphopenic period in early stages posttransplantation and in vitro assessment of alloresponsiveness demonstrated Treg-mediated immunoregulation. This unresponsiveness state persisted beyond 18 months posttransplantation, but no significant Treg-dependent effect was detected afterwards, and the authors concluded that clonal anergy or deletion was the most likely implicated mechanisms in later stages posttransplantation. This protocol appeared to be safe, as no serious opportunistic infections were reported despite prolonged lymphopaenia. However, one patient required treatment for acute antibody-mediated rejection (AMR).

Scholars and researchers of immune tolerance have long-realised that resetting the immune system through systemic and thymic immunoablation followed by BMT has been a successful model for the induction of tolerance in vivo in different experimental models, and this small case series indeed boosts our confidence in such protocols for clinical application. However, wider experience on the safety of immunoablation and combined kidney and BMT as therapy for patients with kidney failure, without haematological malignancies warranting BMT, are still required before wide applicability of such protocols can be recommended to the vast population of kidney failure patients.

## 5. Regulatory T Cells as Immunodiagnostic Tools in Kidney Transplantation

The role of circulating Tregs in the inhibition of allospecific indirect pathway was first reported in 2003 in kidney transplant patients with stable kidney function [48]. It was then hoped that the presence of Tregs or the detection of Foxp3 transcripts in blood, fluid, or tissues, in particular inside the kidney graft, could be reflective of immunoregulation and be correlated with graft outcomes. In the following subsections, we describe the studies assessing the role of the detection of Tregs in the immunodiagnosis of kidney transplant rejection and the prediction of graft outcomes.  Table 1 compiles the studies supporting a role of Treg and/or Foxp3 analysis for the immunodiagnosis of transplant rejection or the prediction of graft outcomes in kidney transplantation, while Table 2 summarises the studies reporting conflicting results or questioning the utility of Tregs and Foxp3 as immunodiagnostic and immunopredictive tools for kidney transplantation. We conclude this section by attempting to reconcile the contradictory results and suggest some conclusions based on the most compelling evidence. 

### 5.1. Studies Associating Regulatory T Cells with the Diagnosis of Kidney Transplant Rejection and with Good Graft Outcomes

Attempting to find a protective value of the detection of Foxp3 transcripts, a prospective study in 36 kidney transplant patients undergoing acute rejection reported significantly higher levels of Foxp3 transcripts in spun urinary cells, associating the presence of Foxp3 with rejection [49]; contrary to the general expectations that expression of Foxp3, being a marker of Tregs, should be lower in rejection. Nonetheless, high Foxp3 transcripts levels were significantly associated, though weakly, with better serum creatinine levels measured during the episode of rejection. In addition, patients with both rejection and higher levels of urinary Foxp3 transcripts had better responsiveness to steroid treatment than the patients with lower levels, and they also had significantly lower risk for graft failure. These authors went on to show that the measurement of Foxp3 transcripts appeared to correlate better with outcomes than the histological grade of the Banff classification. These findings seem to imply that the greater the number of Tregs recruited in response to the inflammation of transplant rejection, the better the chances to overcome it. Moreover, these interesting results suggested a potential role of the measurement of Foxp3 transcripts in the immune diagnosis of kidney transplant patients, and a beneficial link between the presence of Tregs and better kidney transplant outcomes following a rejection episode.

A more recent but small prospective study further supported the relationship of Foxp3 transcripts with rejection [50]. These authors found that Foxp3 transcript levels increased in the peripheral blood of 10 recipients of living-donor kidney transplants undergoing acute rejection in comparison with stable patients.

Another small retrospective study in 17 kidney transplant patients claimed that graft infiltration with Tregs might correlate with favourable graft outcomes [51]. The patients with no Foxp3^+^ T cells within their infiltrates (*n* = 6) had a nonsignificant trend towards higher serum creatinine levels and a significantly higher rate (50%) of graft loss within the first year after transplantation, compared to the patients with Foxp3^+^ T cell infiltrates. Although encouraging, the sample size was too small to be conclusive. Furthermore, 2 of the 3 patients who lost their graft had positive staining for the complement derivative C4d, which is a surrogate marker for antibody-mediated injury, indicating that graft loss could have been due to AMR and not purely due to the lack of Tregs.

### 5.2. Regulatory T Cells Analysis in Delayed Graft Function

In another prospective study of 35 kidney transplant patients with delayed graft function (DGF), a similar conclusion was drawn on the diagnostic potential of Foxp3 expression for acute rejection [52]. The authors reported that the expression of genes associated with CTLs, which are key players in rejection, as well as Foxp3 expression, as a marker of immunoregulation mediated by Tregs, in kidney biopsy tissue, peripheral blood cells, and urinary cells, was significantly higher in patients with acute rejection when compared with those with acute tubular necrosis. Remarkably, the correlation of Foxp3 transcripts with acute rejection was the strongest among all the biomarkers studied, suggesting a utility of reverse-transcriptase polymerase chain reaction for Foxp3 for the early diagnosis of acute rejection in patients with DGF [52].

### 5.3. Regulatory T Cells Role in Borderline Rejection

In a cross-sectional study of biopsies of 15 kidney transplant patients with clinical dysfunction and borderline rejection, higher ratios of transcripts for Foxp3 when compared to transcripts for granzyme B, a surrogate marker for CTLs, were found, in comparison with patients with type IA acute T-cell-mediated rejection (TCMR) [53]. The same authors, in another retrospective study of 46 kidney transplant biopsies with borderline rejection, found that Foxp3 transcripts levels were significantly higher in the 25 patients with stable serum creatinine levels when compared to the 21 patients who progressed to biopsy proven acute rejection (BPAR) in a repeat biopsy at 40 days [54]. The authors did not observe significant difference between the two groups in the expression of granzyme B, IFN*γ*, IL-23, or ROR*γ*t, considered markers of CTLs and Th17 cells. All the patients with BPAR in the repeat biopsy received antirejection treatment, and no difference in kidney function at 2 years was found between this group and the patients who did not progress to BPAR.

Altogether, their findings suggested a protective role of Tregs in ameliorating transplant inflammation and, perhaps, “retarding the progression" in the Banff classification. In addition, their results suggested that the Foxp3 levels in patients with borderline rejection can have a prognostic value, and low levels could warrant a follow-up biopsy or the initiation of antirejection therapy.

### 5.4. Regulatory T Cells Utility in Protocol Biopsies and Subclinical Rejection

In another study, a state of hyporesponsiveness related to peripheral and tissue infiltrating Tregs was observed in kidney transplant patients, particularly with concurrent anti-thymocyte globulin (ATG) induction and sirolimus-based immunosuppressive regimen [55], both known to have positive effects on Tregs (see below). These authors subsequently published interesting findings on a retrospective study on protocol biopsies at 6 months posttransplantation in 37 kidney transplant patients [56]. The presence of Tregs in the infiltrates as well as the proportion of Tregs over the total T cell infiltrate positively correlated with better graft function at 2 or 3 years posttransplantation, regardless of the use of sirolimus, which is known to have positive effects on Tregs. They also proposed that dominant Treg infiltration could be the underlying factor to prevent progression from subclinical rejection (SCR) to clinical rejection.

In a more recent retrospective study of 37 patients diagnosed with SCR in 6-month protocol biopsies by the same group, they corroborated that the presence of Tregs in patients with SCR associates with better kidney function and graft survival up to 5 years posttransplantation, when compared to patients with SCR but with no infiltrating Tregs [18]. This emphasises that using the Banff classification alone may be insufficient for prognostication purposes, and additional characterisation of the infiltrate in kidney transplant biopsies may have added importance. Since the decision to treat patients with SCR is often a therapeutic dilemma, it might be more sensible to treat rejection or to enhance the immunosuppression for those patients with SCR devoid of Treg infiltration.

In another retrospective study of selected 125 protocol biopsies with lymphocyte infiltrates, 14 of them with SCR, 32 of them with borderline rejection, and 79 not classified as rejection, the authors found that the ratio of Foxp3 to granzyme B transcripts was able to predict risk for rejection within one year of biopsy and observed better kidney function and graft survival up to 5 years of followup [57]. Extra 45 protocol biopsies not showing lymphocyte infiltration were not factored in their prediction analysis, but 13% of this subset developed acute rejection within 3 years of followup. Overall their results suggested that when a T-cell infiltrate is present in a protocol biopsy the greater the Treg infiltrate over the Teff infiltrate, the greater the likelihood for better kidney graft outcomes; however, it is difficult to lead to conclusions regarding the utility of the analysis of the Treg/Teff ratio in the absence of lymphocytic infiltrates.

### 5.5. Regulatory T Cells in Chronic Kidney Transplant Rejection

In a retrospective study in 67 biopsies performed due to rising serum creatinine levels, 34 of them showing acute TCMR and 33 displaying chronic TCMR, the ratio of Tregs over total T cells and the absolute number of Tregs was greater in the biopsies with chronic infiltrates in scarred areas than in patients with acute TCMR [58]. In addition, greater numbers of Tregs in inflamed scarred areas appeared to correlate with better graft survival [58]. This study further supports the assessment of total inflammation scores in kidney transplant biopsies and, importantly, suggests that a characterisation of the phenotype of the T cell infiltrates is required to give a more precise prognostic value to a biopsy showing chronic TCMR.

### 5.6. Conflicting Reports on the Association of Regulatory T Cells with Acute Kidney Transplant Rejection and Graft Outcomes Prediction

All the aforementioned studies support a potential clinical utility of Foxp3 expression in the identification of acute rejection and/or the prediction of better graft outcomes. However, contradictory results have also been reported by some other groups.

Despite an apparent association with the diagnosis of rejection, Foxp3 expression did not associate with kidney graft outcomes in a retrospective study of 83 biopsies, 42 of them with BPAR, taken from 77 kidney transplant patients [59]. In their univariate analysis, the authors observed higher expression levels of Foxp3 in patients with acute TCMR and AMR, as well as a correlation with more severe interstitial inflammation, tubulitis, C4d deposition, tubular atrophy, and interstitial fibrosis, but there was no relationship with glomerulitis or arteritis, nor chronic glomerulopathy or vasculopathy. However, in their multivariate analysis only C4d positivity and biomarkers of inflammation were associated with graft outcomes. Thus, the authors concluded that Foxp3 expression was not a marker of alloimmunity but rather of any inflammatory process accompanying acute or chronic rejection [59]. Nevertheless, more detailed studies with a larger sample size per subgroup were suggested by the researchers to confirm these data.

Similar discouraging observations were reported in a relatively large retrospective series on biopsies from 73 kidney transplant patients, 59 of them presenting with BPAR [60]. These authors showed that high expression of Foxp3 was closely associated with acute TCMR, but not with AMR or calcineurin inhibitor (CNI) nephrotoxicity. However, the 2-year graft survival in the kidney transplant patients with acute rejection and high expression of Foxp3 was worse than in those with rejection and low levels of Foxp3. In addition, the authors found no association between the Foxp3 expression level and the serum creatinine level at the time of biopsy. In their immunostaining, they observed that most of the cells expressing Foxp3 were CD4^+^ T cells, while few of them were CD8^+^. One simplistic explanation of the negative association of Foxp3 expression with graft outcomes in this study is that a high observed number of recruited Tregs in the transplant kidney may be a part of an intense inflammatory infiltrate of alloreactive Teffs without having the actual Treg/Teff ratio favouring the immunoregulatory branch of the immune system. Thus, a high number of Teffs would be more difficult to suppress irrespective of a large accompanying Treg infiltrate. Alternatively, the Tregs recruited during a rejection episode could be dysfunctional, so their presence might not correlate with the degree of inflammation or the outcome of the immune activation. Another possibility is that the use of Foxp3 expression as a surrogate marker for Treg presence or function could lead to an overestimation of the actual amount of Treg-mediated immunosuppression, as it is well known in humans Foxp3 can be expressed transiently by Teffs during their activation [19].

Another retrospective study found that the proportion of Tregs in CD4^+^ T infiltrates was higher in borderline rejection and SCR when compared with T-cell-mediated rejection, while the proportion of Teffs was equivalent in both situations [61]. Their results indeed suggested a protective role of the presence of Tregs against overt rejection. However, they question the value of identifying Tregs for the diagnosis of rejection, as their presence was not associated with a worse histopathological diagnosis.

 Other recently published studies also found no correlation between Foxp3^+^ Treg infiltrate and graft function. However, their results must be interpreted with caution. One retrospective study on 32 biopsies taken from 23 kidney transplant patients claimed no diagnostic value for acute rejection nor a prognostic value at 1 year [62]. However, the authors included only patients with Banff type I acute TCMR, excluding patients with higher grades of rejection who are likely to have higher T-cell infiltrates and likely different outcomes. Another recent prospective study on 55 kidney transplant patients also showed no correlation between the Foxp3^+^ Treg infiltrates and kidney function at 12 or 24 months posttransplantation by comparing the results obtained from protocol biopsies with the ones obtained in patients with BPAR [63]. However, in their protocol biopsies group, patients with biopsies devoid of significant inflammatory infiltrate and those not diagnostic of acute rejection were all excluded, which could have biased their results and conclusions.

### 5.7. Are We Ready Then for the Use of Regulatory T Cells for the Diagnosis of Kidney Transplant Rejection and Prediction of Graft Outcomes?

In all these studies, the true significance of the presence of Tregs or upregulated Foxp3 in acute rejection episodes could not be exactly ascertained. Conceivably, it could represent a marker of immunoregulation, or, alternatively, simply reflect the massive T-cell activation during a rejection episode since Foxp3 expression is not only confined to Tregs but also known to be upregulated in activated conventional T cells as well [19]. However, the relation of Tregs with better outcomes in some of these studies supports the former hypothesis, which appears to concur with the animal data demonstrating the presence of functionally active Tregs inside tolerated grafts [43].

At least in part, the surprisingly weak correlation between a positive outcome and the presence of Tregs may be due to the negative effects of immunosuppressive drugs in Treg development and function. Indeed, some reports have showed that a few immunosuppressive drugs like ciclosporin A [64] and basiliximab [65, 66] appear to have a negative effect on Tregs in renal transplant recipients, while other drugs favour the induction and maintenance of Tregs, particularly the inhibitors of the mammalian target of rapamycin [64, 67] and ATG [68–70]. Despite knowledge of the specific effects of immunosuppression on Tregs is of crucial importance, a more detailed description of these effects is beyond the scope of this paper, and interested readers may refer to the relevant section in our previous publication [38].

It is also worth mentioning that immunosuppressive drugs can lead to a reduction in total T-cell numbers and depending on specific cell-type sensitivity to particular drugs, their effects will also skew Treg representation within the T-cell populations in the circulation. Therefore, it appears that analysing intragraft Teffs and Tregs may therefore reflect more accurately the state of the immune system during a rejection episode than the analysis of these cells in peripheral blood. Moreover, it is possible that many of the intercellular interactions or soluble signals tuning the balance from rejection towards alloprotection might be occurring within the draining lymph nodes [71]. However, obtaining draining lymphatic tissue for research or diagnostic purposes in clinical kidney transplantation would be technically challenging and perhaps not sensible from the ethical point of view. Therefore, we can only make extrapolations based on a reductionist model. On balance, it is plausible that the more stable nTregs exert their regulatory action directly on APCs or the activated T cells inside secondarily lymphoid tissue, whilst their less stable partners, the iTregs, act on tissue-infiltrating Teffs instead.

Though there are conflicting results on the utility of Tregs for the immune diagnosis and prediction of outcomes in clinical transplantation, we believe that all the evidence presented here on balance favours a positive role of Tregs in the diagnosis of kidney transplant rejection and prediction of graft outcomes. However, at present, neither general nor definitive recommendations can be drawn regarding the detection of Tregs for the diagnosis of acute kidney transplant rejection for modification of immunosuppressive regimens or outcomes prediction. Despite this, it is possible that the immunostaining for Foxp3^+^ Tregs in patients with SCR on protocol biopsies or in patients with DGF might ultimately be useful to identify patients with high risk of rejection, who might benefit from closer follow up or a more tailored immunosuppressive regimen.

## 6. Regulatory T Cells as Immunomodulatory Therapy in Clinical Kidney Transplantation

### 6.1. Paving the Path

There is also ample evidence in the literature demonstrating the potential use of Tregs as therapeutic tools [5]. The seed of hope for Tregs to be used as immunotherapy germinated from adoptive transfer experiments in mice, where the administration of T cells with regulatory properties was shown to prolong the survival of allogeneic grafts [72, 73]. Interestingly, this transplant tolerance can be also “infectiously” transferred to naïve animals by the administration of Foxp3^+^ Tregs from tolerant animals, showing a therapeutic potential [74]. In addition, the transduction of CD4^+^CD25^−^ T cells with the Foxp3 gene, in other words, naïve T cells forcefully converted into iTregs, were associated with the prolongation of the survival of skin allografts in mice [75]. While in principle the presence of Tregs is required to achieve tolerance, functional state and subset composition may be more crucial for long-term graft survival. In a murine adoptive transfer model, it was observed that Tregs from CD39-deficient mice were not efficient in preventing rejection of allogeneic skin grafts in comparison with the administration of wild-type Tregs expressing CD39 [23]. In humans, CD39 is specifically expressed on the T_REM_ subset [24], which suggests a potential role of this subset in an allogeneic transplantation setting and a potential use of the expression of CD39 on Tregs as part of their armamentarium to perform immunoregulation. However, very scarce data exist on the administration of Tregs for the induction of immunosuppression, prevention, or treatment of rejection, especially for solid organ transplantation, in comparison with the advances achieved for haematopoietic stem cell transplantation (HSCT) and the prevention of graft-versus-host disease [76–78]. It may be more difficult to manipulate a complex alloresponse induced by structurally intricate organs like the kidney, in which many cell types and more varied arms of the immune response could be implicated. In addition, unlike HSCT, solid organ transplantation does not benefit from the resetting of the immune system and the microchimerism that usually follow HSCT. Nonetheless, the growing amount of data and protocols used for HSCT is serving as scaffolding for the construction of protocols in solid organ transplantation.

### 6.2. Overcoming the Challenges

Besides the partially understood immunological barriers, there are quite a few technical barriers as well as clinical and ethical concerns that have made implementation of Treg-based therapy an uphill task. Nevertheless, major progress has been made that could give Treg infusions a place in clinical transplantation in the near future. It has been difficult to enrich this relatively rare population of T cells from peripheral blood, from which a definitive surface selection marker has been lacking. Although CD4 and CD25 continue to be useful for the identification of Tregs, neither marker is unique to human Tregs in contrast to mice. Intermediate amounts of CD25 are expressed by conventional Teffs and the T_REM_ subset, and, furthermore, the positive selection of Tregs using anti-CD25 mAbs could alter the activation status of the cells. Improved selectivity could be reached by combining CD25 with CD127, a marker found to be absent on most Tregs [79, 80]. A recently described purification method of Tregs even offers to obtain “untouched” Tregs [81]. This purification method, which involves the removal of CD49d^+^/CD127^+^ cells, containing much of the unwanted CD25^+^ Teffs, leads to enrichment of a pure population of Tregs, which are CD49d^−^/CD127^ low^.

 Tregs also appear to be difficult to expand in culture and to maintain for prolonged periods of time, not to mention their very restrictive culture needs. Nevertheless, several protocols have been developed for large scale expansion of Tregs. For instance, high doses of IL-2 plus anti-CD3/anti-CD28 mAbs or the use of DCs together with anti-CD3 mAbs have been used for polyclonal expansion of Tregs isolated from mouse and human. Not only that the Tregs expanded under these conditions retained their suppressive activity, but also their potency appeared to increase compared to freshly isolated Tregs [36, 82–85]. Artificial APCs have also been used to facilitate the expansion of nTregs in vitro [86]. Another strategy of in vitro conversion of human CD4^+^CD25^−^ non-Treg cells into functional Foxp3^+^ Tregs involves the stimulation of the cells with TGF*β* and sirolimus [87]. However, it is estimated that greater numbers of Tregs might be required to maximise efficacy in solid organ transplantation, probably in the order of 10^9^ to 10^11^, when compared to HSCT [76–78], where fewer Treg numbers appear to be sufficient. Nonetheless, recent protocols have demonstrated the ability to generate and expand large numbers of phenotypically and functionally stable allospecific Tregs [88, 89], a method that could be applied readily in translational medicine.

Finally, knowledge of the pharmacological effects of immunosuppressive drugs on Treg function and development is of outmost importance when designing protocols involving the administration of Tregs, in order to preempt a potentiating or minimising effect.

### 6.3. Is It Antigen Specificity Important?

An important notion extrapolated from animal work in transplantation tolerance is the observation that antigen-specific Tregs and perhaps the most efficient Tregs home to and remain inside secondary lymphoid tissue or inside the graft rather than in the circulation. In animals, purifying Tregs from these compartments is feasible, but not in the clinical setting. Thus, purifying Tregs from peripheral blood for their use in transfer protocols could lead to the enrichment of less efficient non-antigen-specific Tregs, but this is still our compromise. This can have practical implications as antigen-specific Tregs appear to be more powerful to perform immunoregulation than their polyclonal counterparts [89]. It is also possible that the in vitro manipulation of Tregs might alter their migratory capacities, impeding their desired migration to the allograft or its draining lymph nodes, but it has been demonstrated that ex vivo expanded Tregs can locate inside the graft and lymph nodes [88, 89], where they should be able to perform immunoregulation. The creation of a frozen nTreg cell bank to permit Treg administration to different patients or to be used if recurrent maintenance Treg infusions are required would be of practical use. However, cryopreservation of Tregs is also not an easy task, as it can affect the yield and health of the cells. In this respect, the cryopreservation of donor-specific Treg clones will be more feasible in recipients of living donor kidney grafts than in deceased donor transplantation, where the transplant surgery occurs in an emergency basis and it is practically impossible to generate donor-specific Tregs.

 It is worth mentioning that donor-specific Tregs have been generated in vitro with either direct or indirect allorecognition capacity. The direct pathway of allorecognition involves the stimulation of T cells with intact allogeneic MHC molecules presented by donor APCs, the so-called “passenger” leukocytes. The direct pathway is a powerful stimulator of alloresponses, starting as soon as the transplant is grafted into the recipient. With the migration and eventual demise of the “passenger” leukocytes, the cells bearing intact donor-derived MHC molecules, the contribution of the direct pathway in chronic rejection or triggering acute episodes of rejection late in transplantation, might not be as significant as the indirect pathway. On the other hand, the indirect pathway involves the presentation of the allogeneic determinants via the recipient APCs through the self-MHC molecules loaded with the peptides from the allogeneic donor-derived MHC molecules, which is in fact the conventional way of antigen presentation. This indirect pathway is believed to be the most important drive of chronic rejection as depend mainly on the uptake and presentation of the MHC antigens present on the parenchymal cells within the transplant [90, 91], therefore, allogeneic MHC-derived antigens are constantly delivered to the immune system. In fact, administration of Tregs specific for both the direct and the indirect pathway of allorecognition was able to prevent acute rejection episodes as well as chronic rejection changes in a murine model of skin and cardiac transplantation preconditioned with sublethal irradiation and BMT, while Tregs with direct specificity alone were able to protect only against acute rejection but not chronic rejection [41]. It must be emphasised that in this model, BMT alone failed to protect against rejection, illustrating the importance of combining strategies in order to secure a highest success rate for immunoregulatory therapies applicable in the clinical setting.

 From all these observations, it can be concluded that the differential generation of Tregs targeting these two different pathways of allorecognition should provide a practical advantage to target different arms of the alloresponse and at different stages posttransplantation. Thus, we can postulate that donor-specific Tregs with specificity to intact allogeneic MHC molecules [89], the direct pathway, could be used mainly at induction phases of immunosuppression, while Tregs with indirect allospecificity [88] could be used as maintenance adjuvants of immunosuppression.

### 6.4. Potential Hazards of Regulatory T-Cell Administration

Another concern, given that Tregs appear to perform their effector regulatory mechanisms in an antigen-unspecific way, the systemic administration of Tregs could cause unwanted bystander inactivation of required immune responses against viruses or other pathogens, as well as for immunosurveillance against cancer. However, no increased risk for these potential side-effects were observed in the published trials on HSCT [76–78]. In addition, the use of allospecific Tregs should minimise even further such potential risks. A notable observation that has troubled our minds is the transient expression of Foxp3 by some human Teffs upon activation [19]. This could lead to the enrichment of undesired Teffs if Foxp3 expression is used as the Treg-identification marker or surrogate marker of Treg function, with the risk of adoptively transferring contaminant Teffs that likely could attack the graft rather that prevent an immune rejection. Similarly, Tregs may display phenotypic plasticity when in contact with proinflammatory conditions [92, 93]. They have been shown to be able to transform into pathogenic alloreactive T cells with a Th17-like phenotype, which brings the danger of sensitising the recipient instead of promoting tolerance if the injected Tregs were to lose their phenotype once in contact with the host immune system [94]. But the ability to maintain stable functional Tregs for prolong period of times and being able to perform immunoregulation in animal models have been published [88, 89] and opens up bright perspectives.

### 6.5. Are We Ready Then for the Use of Regulatory T Cells for the Immunotherapy of Kidney Transplantation?

At present, there is no published evidence on the use of Tregs for solid organ clinical transplantation. There are many technical, logistic and ethical barriers to be faced by researchers interested in Treg-based immunotherapy. Despite encouraging results and promising protocols and given the complexity of the immune system and the diversity of the mechanisms involved in self-tolerance, it is unlikely that the sole administration of Tregs could successfully lead to permanent engraftment of an organ in the absence of immunosuppression. So, we foresee Treg-therapy, at least at present, as an adjunctive potential therapy. Nevertheless, a couple of promising trials have been started on the use of Treg-based therapy for kidney transplantation, and a few more are expected to surface in coming years. A Russian group led by Kaabak and Bykovskai is, at time of writing, performing a clinical trial testing the role of Treg infusions on kidney transplantation in children. In their protocol, ex vivo expanded autologous Tregs are administered at months 1 and 6 posttransplantation in patients on concurrent immunosuppression regimen based on alemtuzumab, a CNI and mycophenolate mofetil. Similarly, the ONE study involves a large effort from four European countries and the United States of America to assess the use of nTreg infusions (among other cells with regulatory capacities) as adjunctive therapy of immunosuppression in kidney transplantation. The outcomes of these trials would be much anticipated. Finally, it is possible that future alternatives with greater likelihood of inducing graft tolerance could include hybrid therapies combining Treg administration with immunoablation and combined kidney and BMT.

## 7. Concluding Remarks

It seems to be of importance to explore an association between certain Treg:Teff profiles with clinical parameters, biomarkers of kidney injury or tolerance, kidney biopsy findings, and clinical outcomes in kidney transplantation. Although there are contradictory reports on the utility of Tregs for the immune diagnosis and prediction of outcomes in clinical kidney transplantation, we believe that there is more compelling evidence supporting the role of Tregs in the immune diagnosis of acute kidney transplant rejection and outcomes prediction. In particular, characterisation and quantification of Tregs in patients with SCR on protocol biopsies or patients with DGF may be useful to help us to tailor their immunosuppressive regimens, but further studies validating such observations should be performed.

 Study outcome of the ONE study, the largest international trial on the use of Treg-based therapy in adult kidney transplantation, is enthusiastically awaited. However, before we can use Treg infusions as routine prescriptions in the wards, few technical difficulties in generating large numbers of a highly pure population of autologous Tregs have to be resolved. To be used in the clinical setting, these Tregs should be allospecific and possess a stable Treg phenotype with prime regulatory properties, so as to avoid excessive bystander suppression and potential undesired predisposition to infections or neoplasias.

 Contrary to the commonly held belief, it appears that Tregs may not be a definitive marker for long-term engraftment and/or graft tolerance and might not be “the clue” in our search for tolerance. Although it seems a long way to go in finding the desperately-awaited “holy-grail,” the crusade for transplantation tolerance shall never cease.

##  Conflict of Interests

There is no conflict of interests among the authors or the participating institutions.

## Figures and Tables

**Table 1:** Studies supporting the association of regulatory T cells with diagnosis of kidney rejection and graft outcomes prediction.

Reference	Study design	Patient characteristics	Immunodiagnostic findings	Graft outcomes	Conclusions/additional comments
Muthukumar et al. [49]	Prospective	83 total pts (36 pts with graft dysfunction and BPAR)	Increased Foxp3 transcripts in urinary cells in pts with BPAR	Better kidney function and lower risk of graft failure in pts with BPAR and higher levels of Foxp3 transcripts	Demonstration of potential role of the measurement of Foxp3 transcripts for diagnosis of rejection and outcomes prediction

Wang et al. [50]	Prospective	10 living-donor transplant pts with BPAR	Increased Foxp3 transcripts in peripheral blood associated with BPAR		Measurement of Foxp3 transcripts in peripheral blood may aid the immunodiagnosis of rejection in living-donor transplantation/sample size too small to be conclusive, but findings are encouraging

Martin et al. [51]	Retrospective	11 of 17 pts with acute BPAR		Patients with Foxp3^+^ infiltrates had better kidney function and lower rates of graft loss at 1-year posttransplantation	Tregs infiltration in graft might correlate with favourable graft outcomes/sample size too small to be conclusive and some patients who lost their graft had evidence of humoral rejection

Aquino-Dias et al. [52]	Prospective	35 pts with DGF (total 48 biopsies: 20 with BPAR and 28 with ATN)	Increased Foxp3 transcripts in kidney tissue, peripheral blood and urinary cells correlated with BPAR when compared to ATN		Measurement of Foxp3 transcripts may aid the early identification of rejection for pts with DGF

Grimbert et al. [53]	Cross-sectional	26 pts with kidney dysfunction (15 pts with borderline rejection and 11 with type IA acute TCMR)	Higher ratios for the transcripts of Foxp3/granzyme B were found in pts with borderline rejection in comparision with pts with type IA acute TCMR		Tregs could play a protective role ameliorating inflammation and preventing the progression to frank rejection

Mansour et al. [54]	Retrospective	46 pts with borderline rejection	Increased Foxp3 transcripts in pts with stable creatinine compared to pts who progressed to BPAR	No difference found in kidney function at 2 years in both pts with treated BPAR and pts with stable creatinine	Measurement of Foxp3 transcripts may have prognostic value in borderline rejection

Bestard et al. [56]	Retrospective	37 pts with SCR from 170 protocol biopsies	Fewer Tregs were observed in pts on ciclosporin	The presence of Treg infiltrates correlated with better graft function at 2- and 3-years posttransplantation	Presence of Tregs infiltration could prevent the progression from SCR to clinical rejection

Bestard et al. [18]	Retrospective	37 pts with SCR	Number of infiltrating Tregs in pts with SCR correlated with Foxp3 demethylation at the Treg-specific demethylation region	The presence of Treg infiltrates correlated with better graft function and survival up to 5-year posttransplantation	Presence of Tregs infiltration in rejection associates with better graft outcomes/the Banff classification alone is insufficient for immunodiagnosis and prognostication purposes

Xu et al. [57]	Retrospective	125 pts with protocol biopsies with presence of T cell infiltrates (14 with SCR, 32 with borderline rejection, 79 with no rejection)	Higher ratios for the transcripts of Foxp3/granzyme B correlated with lower risk for rejection	Higher ratios for the transcripts of Foxp3/granzyme B predicted better kidney function and graft survival up to 5-year posttransplantation	Tregs could play a protective role reducing rejection risk and improving graft outcomes/unable to provide conclusions for pts without T cell infiltrates

Zuber et al. [58]	Retrospective	67 pts (34 with acute TCMR, 33 with chronic TCMR)	Higher number and ratio of Tregs over total T cells were observed in patients with chronic rejection than with acute rejection	The greater the Treg infiltrate in inflamed scarred areas, the better the graft survival	Tregs might be protective in chronic rejection

ATN: acute tubular necrosis; BPAR: biopsy proven acute rejection; DGF: delayed graft function; pts: patients; SCR: subclinical rejection; TCMR: T-cell-mediated rejection. Blank area: not assessed.

**Table 2:** Studies with conflicting results regarding the association of regulatory T cells with kidney rejection and graft outcomes prediction.

Reference	Study design	Patient characteristics	Immunodiagnostic findings	Graft outcomes	Conclusions/additional comments
Bunnag et al. [59]	Retrospective	77 pts (42 with BPAR)	Higher levels of Foxp3 transcripts associated with acute TCMR and AMR in the univariate but not in the multivariate analysis	No relationship was found between Foxp3 expression and graft outcomes. Only C4d positivity and inflammation biomarkers related to outcomes in their multivariate analysis	Foxp3 expression accompanies the inflammatory process rather than being a marker of alloimmunity

Veronese et al. [60]	Retrospective	73 pts (59 with BPAR)	High expression of Foxp3 was associated with acute TCMR but not with AMR	2-year graft survival was worse in pts with BPAR and higher Fopx3 expression	No prognostic value was given to the analysis of Foxp3 expression in patients with BPAR/differentiation of authentic Tregs from recently activated Foxp3^+^ T cells could have been useful to understand their results

Taflin et al. [61]	Retrospective	24 biopsies with graft dysfunction (12 with BPAR and 12 with borderline rejection) and 16 protocol biopsies at 1-year posttransplantation	Treg infiltrates were higher in borderline rejection and SCR compared to patients with acute TCMR		Tregs may have a beneficial role against overt rejection. The authors questioned the diagnostic value of Treg identification for the diagnosis of rejection

Batsford et al. [62]	Retrospective	32 biopsies taken on 23 pts (16 biopsies with BPAR)	No relation of Foxp3^+^ Tregs detection with acute rejection	No prognostic value of the measurement of Tregs at one-year posttransplantation	Measurement of Tregs has no diagnostic nor prognostic value/only Banff type 1 acute TCMR was included, and biopsies with higher grades of rejection and likely larger infiltrates were excluded, which likely biased their results

Kollins et al. [63]	Prospective	55 pts (29 protocol biopsies with no rejection, and 26 indication biopsies with BPAR)	No association between numbers of infiltrating Tregs and diagnosis of rejection	No correlation between Treg infiltrates and kidney function at 1- and 2-year posttransplantation	Measurement of Tregs has no diagnostic nor prognostic value/in the protocol biopsy group, biopsies devoid of significant inflammatory infiltrate and those with infiltrate not diagnostic of acute rejection were excluded, which likely biased their results

AMR: antibody-mediated rejection; BPAR: biopsy proven acute rejection; pts: patients; TCMR: T-cell-mediated rejection. Blank area: not assessed.
